# A survey on antimicrobial resistance genes of frequently used probiotic bacteria, 1901 to 2022

**DOI:** 10.2807/1560-7917.ES.2023.28.14.2200272

**Published:** 2023-04-06

**Authors:** Adrienn Gréta Tóth, Maura Fiona Judge, Sára Ágnes Nagy, Márton Papp, Norbert Solymosi

**Affiliations:** 1 Centre for Bioinformatics, University of Veterinary Medicine Budapest, 1078 Budapest, Hungary

**Keywords:** antimicrobial resistance, resistome, mobilome, probiotic strains

## Abstract

**Background:**

Antimicrobial resistance (AMR) is caused by AMR determinants, mainly genes (ARGs) in the bacterial genome. Bacteriophages, integrative mobile genetic elements (iMGEs) or plasmids can allow ARGs to be exchanged among bacteria by horizontal gene transfer (HGT). Bacteria, including bacteria with ARGs, can be found in food. Thus, it is conceivable that in the gastrointestinal tract, bacteria from the gut flora could take up ARGs from food.

**Aim:**

The study objective was to gain insight into the ARG set carried by commonly used probiotic bacteria that may enter the human body with non-fermented foods, fermented foods, or probiotic dietary supplements (FFPs) and to assess ARG mobility.

**Methods:**

Next generation sequencing whole genome data from 579 isolates of 12 commonly employed probiotic bacterial species were collected from a public repository. Using bioinformatical tools, ARGs were analysed and linkage with mobile genetic elements assessed.

**Results:**

Resistance genes were found in eight bacterial species. The ratios of ARG positive/negative samples per species were: *Bifidobacterium animalis* (65/0), *Lactiplantibacillus plantarum* (18/194), *Lactobacillus delbrueckii* (1/40), *Lactobacillus helveticus* (2/64), *Lactococcus lactis* (74/5), *Leucoconstoc mesenteroides* (4/8), *Levilactobacillus brevis* (1/46), *Streptococcus thermophilu*s (4/19). In 66% (112/169) of the ARG-positive samples, at least one ARG could be linked to plasmids or iMGEs. No bacteriophage-linked ARGs were found.

**Conclusion:**

The finding of potentially mobile ARGs in probiotic strains for human consumption raises awareness of a possibility of ARG HGT in the gastrointestinal tract. In addition to existing recommendations, screening FFP bacterial strains for ARG content and mobility characteristics might be considered.

**Key public health message**

**What did you want to address in this study?**

Antimicrobial resistance (AMR) challenges treating infections. In bacteria, AMR relies on antibiotic resistance genes (ARGs), some of which can be mobile. In certain conditions, bacteria with mobile ARGs may transfer their ARGs to other bacteria. If bacteria with mobile ARGs are in food, they may upon ingestion pass on these ARGs to the bacteria that are present in the digestive tract of humans. We wished to shed light on ARGs in probiotic bacterial species, particularly their mobility characteristics.

**What have we learnt from this study?**

Among 12 probiotic species of interest, we analysed in detail 10 species commonly used in non-fermented/fermented foods or probiotic dietary supplements (FFPs). Using bioinformatics, we screened their genetic data for ARGs and then assessed if the ARGs were mobile. Overall, several types of ARGs were found. Their occurrence varied between species, with no ARGs detected in two species. Among samples of bacteria with ARGs, a considerable proportion had ARGs that were likely mobile.

**What are the implications of your findings for public health?**

Eating foods that contain bacteria with mobile ARGs may allow such bacteria to come near other bacteria found in the human body. This proximity could facilitate mobile ARGs’ transfer from the food bacteria to other bacteria in the gut, even pathogenic ones. While acquiring mobile ARGs does not always confer AMR, extending current recommendations to detect potential functional traits of concern in bacteria used for food might be considered, with screening for mobile ARGs in probiotic bacteria.

## Introduction

Antimicrobial resistance (AMR) is one of the foremost threats hindering the treatment of infectious diseases worldwide, both in human and animal medicine. Due to the clear relatedness of excess antimicrobial use (AMU) and elevated AMR rates, measures are required on a permanent basis. Despite the interventions to reduce AMU, high levels of antibiotic consumption in both animals and humans are still being reported in several countries [1]. It is currently estimated that 700,000 people are dying per annum from AMR-related issues worldwide, with projections forecasting this number to rise to 10 million by 2050 [2]. Identifying potential sources of AMR is thus of utmost importance. AMR can be acquired by bacteria via gene mutations or horizontal gene transfer (HGT) [3]. HGT occurs primarily by transformation, conjugation, or transduction and involves small fragments of DNA being transferred between bacteria [4]. The transfer of AMR genes (ARGs) is enhanced through bacteriophages, integrative mobile genetic elements (iMGEs) and plasmids [5,6].

In our previous work based on metagenomics, and in agreement with the results of other research groups [7-11], we found that non-fermented [12] and fermented [13] foods or probiotic dietary supplements [14] contain a considerable number of ARGs, some of which are mobile. When bacteria with mobile ARGs are consumed through these foods, the ARGs can enter the digestive tract, where it is conceivable that they may be transferred to non-pathogenic bacteria and facultative pathogenic bacteria. This process might even be facilitated if the ingested bacteria are prone/able to colonise the intestinal tract. Since probiotic bacteria are expected to colonise the gut, their AMR determinants can contribute to the gut resistome [15-17].

The aim of our study was to gain insights into the set of ARGs and their mobility potential in prominent probiotic bacterial strains (from the Bifidobacteriales and Lactobacillales order) isolated from food (fermented and non-fermented) and probiotics using a unified bioinformatic pipeline.

## Methods

Our work is based on next generation sequencing (NGS) data from isolates of 12 commonly used probiotic bacterial species that have been isolated in other studies. The bacterial species were selected based on a non-systematic review. This involved a search of the PubMed database (https://pubmed.ncbi.nlm.nih.gov/) using the keywords ’kefir’, ’yogurt’, ’probiotic’, and ’bacteria’, for papers published in English after 2000. From the hits, we selected reports with relevant data (see below) on probiotic bacterial species [18-23].

### Data used

Data that met the following criteria: having genomic library source, being whole genome sequenced and being Illumina platform based were downloaded from the National Center for Biotechnology Information (NCBI) Sequence Read Archive (SRA) repository.

The selected species (and sample download dates in day/month/year) were: *Bifidobacterium animalis* (4/12/2022), *Lacticaseibacillus casei* (4/12/2022), *Lacticaseibacillus paracasei* (4/12/2022), *Lactiplantibacillus plantarum* (3/12/2022), *Lactobacillus delbrueckii* (25/12/2022), *Lactobacillus helveticus* (4/12/2022), *Lactobacillus kefiranofaciens* (25/12/2022), *Lactobacillus kefiri* (25/12/2022), *Lactococcus lactis* (6/12/2022), *Leucoconstoc mesenteroides* (25/12/2022) *Levilactobacillus brevis* (2/12/2022), *Streptococcus termophilus* (3/12/2022).

The source data collected on samples were grouped into three categories: non-fermented food or fermented food or probiotic (FFP) bacteria, intestinal bacteria, and others. The FFP group included samples with the following origins: fermented beverage (n = 12), fermented food (n = 69), fermented food of dairy nature (n = 124), fermented food of non-dairy nature (n = 141), milk with source non-specified (n = 10), milk from farm animal (n = 14), milk of human origin (n = 34), milk/dairy product (n = 26), probiotic dietary product (n = 1), probiotic dietary supplement (n = 146), starter culture (n = 2). The detailed metadata for each sample are available at: https://doi.org/10.6084/m9.figshare.21877134.v2.

### Bioinformatic and statistical analysis

Quality based filtering and trimming of the raw short reads was performed with TrimGalore (v.0.6.6, https://github.com/FelixKrueger/TrimGalore), setting 20 as a quality threshold. Only reads longer than 50 bp were retained.

Cleaned reads from each bacterial species were aligned to the representative reference genome for the corresponding bacterium species (*B. animalis*: NC_017216.2; *L. casei*: NZ_AP012544.1; *L. paracasei*: NC_022112.1; *L. plantarum*: NZ_CP028221.1; *L. delbrueckii*: NZ_CP018218.1; *L. helveticus*: ASM2283254v1; *L. kefiranofaciens*: NZ_CP061341.1; *L. kefiri*: NZ_CP029971.1; *L. lactis*: NZ_CP059048.1; *L. mesenteroides*: NZ_CP028251.1; *L. brevis*: NZ_CP015398.1; *S. thermophilus*: NZ_LR822015.1) for each sample by Bowtie2 [24]. Reads from samples that covered at least 80% of the respective reference genome were de novo assembled with MEGAHIT (v1.2.9) [25] using default settings.

From the contigs all possible open reading frames (ORFs) were gathered with Prodigal (v2.6.3) [26]. The protein translated ORFs were aligned to the ARG sequences of the Comprehensive Antibiotic Resistance Database (CARD, v.3.2.5) [27,28] by Resistance Gene Identifier (RGI, v6.0.0) with Diamond [29]. The ORFs classified as perfect or strict were further filtered with 90% identity and 90% coverage. Moreover, it was taken into account that when the bit-score of a hit is below the gene-specific predetermined cut-off value while the identity exceeds the 95% threshold, the RGI for ARG annotation classifies the hit as strict and uses the nudged notation. As a resistance gene prediction, which is a nudged, is more prone to be a false positive result, all nudged hits were excluded.

The iMGE content of contigs harbouring ARGs was analysed with MobileElementFinder (v1.0.3) and its database (MGEdb v1.0.2) [6]. Following the distance concept of Johansson et al. [6], an ARG was considered to be associated with an iMGE if it was within a given distance of this iMGE. In the MGEdb we found data only for *L. lactis*, the longest composite transposon (cTn) for that species was the *Tn5721*, its length (11,256 bp) was taken as the cut-off value. For the rest of the species, a general threshold value was declared as the median of the longest cTns per species in the database (10,098 bp). The plasmid origin probability of the contigs was estimated by PlasFlow (v.1.1) [30]. The phage content of the assembled contigs was predicted by VirSorter2 (v2.2.1) [31]. The findings were filtered for dsDNAphages and ssDNAs. For MobileElementFinder, PlasFlow and VirSorter2 the default settings were used.

The 95% confidence intervals (CI) were estimated using the exact method for prevalence [32]. All data management procedures, analyses and plots were performed in R environment (v4.2.1) [33].

## Results

### Sample characteristics

For the 12 species, a total of 2,244 samples were downloaded. After fitting to the reference genomes, 1,452 of these samples were retained, as the obtained sequence covered at least 80% of the reference genome. Of these, 579 samples were from FFP isolates, 559 had intestinal origin and 314 originated from other sources. The data by sample is presented separately (https://doi.org/10.6084/m9.figshare.21877134.v2).

In non-FFP samples, *L. kefiranofaciens* (n = 5 samples) and *L. kefiri* (n = 2 samples) species were present. In contrast, no *L. kefiranofaciens* or *L. kefiri* species could be observed in the FFP samples. The 579 FFP samples comprised the following species: *B. animalis* (n = 65 samples), *L. casei* (n = 1 samples), *L. paracasei* (n = 33 samples), *L. plantarum* (n = 212 samples), *L. delbrueckii* (n = 41 samples), *L. helveticus* (n = 66 samples), *L. lactis* (n = 79 samples), *L. mesenteroides* (n = 12 samples), *L. brevis* (n = 47 samples) and *S. thermophilus* (n = 23 samples).

**Figure 1:**
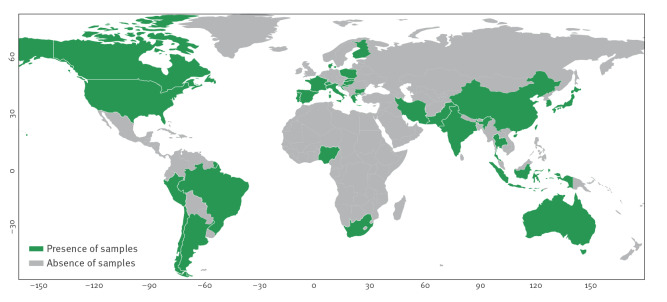
Geographical origin of bacterial samples from food or fermented food or probiotics analysed the study, 1901–2022 (n = 561 samples)^a^

^a^ For 18 samples, the country of origin was not available.

All FFP samples with available collection date had been obtained between 1901 and 2022. Eight of the 579 samples had no year recorded for the time of their collection. In contrast, none of the samples had the release date missing. The release dates of the whole genome sequenced data were between 10 February 2014 and 15 November 2022. For 561 of the FFP samples, the country of origin was described. The countries from which samples originated are shown in Figure 1 and Table 1. 

**Table 1:** Proportions of ARG-positive bacterial samples^a^ found in the study, stratified by country, 1901–2022 (n = 561 isolates)^b^

Country	Number of samples with ARGs /total number of samples	Country	Number of samples with ARGs /total number of samples
Argentina	6/10	Iran	0/3
Australia	1/20	Italy	6/8
Brazil	0/1	Japan	0/3
Bulgaria	0/3	Korea	0/2
Canada	0/2	Nigeria	0/3
Chile	0/2	Pakistan	0/1
China	60/198	Peru	3/3
Croatia	2/10	Poland	2/2
Denmark	3/4	Portugal	0/2
Finland	1/1	Slovakia	0/14
France	1/2	South Africa	0/1
Greece	0/7	Spain	4/5
Hungary	0/1	Switzerland	4/6
India	4/10	Thailand	0/1
Indonesia	0/3	United States	67/232

ARG: antimicrobial resistance gene.

^a^ Samples were from food or fermented food or probiotics.

^b^ For 18 samples, the country of origin was not available, five of which were ARG-positive. In one sample from Taiwan, no ARG was detected.

### Resistome

**Figure 2:**
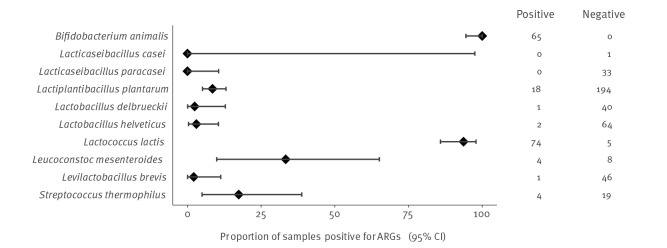
Proportion of ARG positive samples, stratified by species, 1901–2022 (n = 579 isolates)

ARG: antimicrobial resistance gene; CI: confidence interval.

Of 579 FFP samples, 169 were ARG-positive (29%; 95%CI: 26–33). The proportions of ARG-positive samples stratified by their country of origin are presented in Table 1 and by species in Figure 2. The names of the ARGs identified and their number per species are summarised in Table 2. Among the samples analysed, ARGs were neither identified in the single sample of *L. casei* nor in the 33 samples of *L. paracasei* species (Figure 2).

The numbers of ARG positive and negative samples for each species are listed beside the prevalence (diamond) and its 95%CI (horizontal line).

**Table 2:** Description per species, of identified ARGs with the antibiotics they potentially confer resistance to, and resistance mechanisms, 1901–2022 (n = 579 isolates)

Bacterial species (number of samples with ARG (n); total number of samples (N))		ARG (number of samples bearing the ARG)		Antibiotics the ARGs may confer resistance to^a^	Mechanisms of resistance potentially involved (proportion of ARG-containing-samples with a given mechanism)^a^
*Bifidobacterium animalis*	(n = 65; N = 65)	*B. adolescentis rpoB* mutations conferring rifampicin resistance	(n = 65)	Phenicols, rifamycins, tetracyclines	Antibiotic inactivation (1/65); antibiotic target alteration and target replacement (65/65); antibiotic target protection (65/65)
		*Limosilactobacillus reuteri cat-TC*	(n = 1)		
		*tet(W)*	(n = 65)		
*Lactiplantibacillus plantarum*	(n = 18; N = 212)	*AAC(6’)-Ii*	(n = 2)	Aminoglycosides, cephalosporins, lincosamides, macrolides, monobactams, penams, penems, phenicols, pleuromutilins, streptogramins, tetracyclines	Antibiotic efflux (1/18); antibiotic inactivation (14/18); antibiotic target alteration (1/18); antibiotic target protection (10/18)
		*ANT(3”)-IIa*	(n = 6)		
		*ANT(6)-Ia*	(n = 1)		
		*catA8*	(n = 1)		
		*eatAv*	(n = 2)		
		*ErmB*	(n = 1)		
		*lnuA*	(n = 2)		
		*msrC*	(n = 2)		
		*TEM-1*	(n = 1)		
		*TEM-181*	(n = 1)		
		*tet(C)*	(n = 1)		
		*tet(M)*	(n = 5)		
		*tet(S)*	(n = 1)		
*Lactobacillus delbrueckii*	(n = 1; N = 41)	*TEM-116*	(n = 1)	Cephalosporins, monobactams, penams, penems	Antibiotic inactivation (1/1)
*Lactobacillus helveticus*	(n = 2; N = 66)	*lnuA*	(n = 2)	Lincosamides	Antibiotic inactivation (2/2)
*Lactococcus lactis*	(n = 74; N = 79)	*ErmB*	(n = 1)	Lincosamides, macrolides, streptogramins, tetracyclines	Antibiotic efflux (71/74); antibiotic target alteration (1/74); antibiotic target protection (5/74)
		*lmrD*	(n = 71)		
		*tet(M)*	(n = 1)		
		*tet(S)*	(n = 4)		
*Leucoconstoc mesenteroides*	(n = 4; N = 12)	*ANT(3”)-IIa*	(n = 3)	Aminoglycosides, lincosamides	Antibiotic efflux (1/4); antibiotic inactivation (3/4)
		*lmrD*	(n = 1)		
*Levilactobacillus brevis*	(n = 1; N = 47)	*lnuA*	(n = 1)	Lincosamides	Antibiotic inactivation (1/1)
*Streptococcus thermophilus*	(n = 4; N = 23)	*ErmB*	(n = 2)	Lincosamides, macrolides, streptogramins, tetracyclines	Antibiotic target alteration (2/4); antibiotic target protection (2/4)
		*tet(S)*	(n = 2)		

ARG: antimicrobial resistance gene.

^a^ Information on the antimicrobial resistances that the genes may contribute to, and the mechanisms of action originates from the Comprehensive Antibiotic Resistance Database (CARD) database [27,28].

For *Lactiplantibacillus plantarum*, one sample had two ARGs, two had three ARGs and one had four ARGs. For *Lactococcus lactis* three samples had two ARGs. For *Leucoconstoc mesenteroides*, four samples had one ARG. For *Streptococcus thermophilus*, four samples had one ARG*.*

Table 2 also lists the antibiotics that the ARGs may contribute resistance to. All 65 samples of *B. animalis* had 15 (V516E, E525V, D532E, A533V, E543K, K552E, Q554E, A557V, V559D, G560A, E561A, E562G, V565E, S570E, S571M) of the 16 *B. adolescentis rpoB* mutations in the rifampicin pocket, which confer resistance to rifampicin, the only absent mutation being A443V [34].

The resistance mechanisms, that the identified ARGs have previously been shown to be involved in, included antibiotic efflux, antibiotic inactivation, antibiotic target alteration and/or antibiotic target replacement and antibiotic target protection (Table 2).

### Mobilome

**Figure 3:**
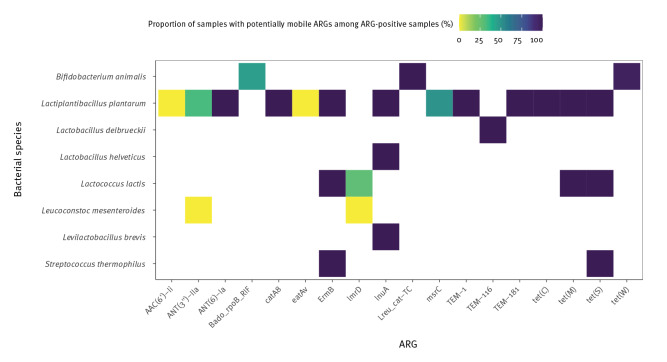
Proportion of samples found with potentially mobile ARGs among ARG-positive samples, 1901–2022 (n = 169 samples)

No ARGs that could be linked to bacteriophages were found in any sample. On the other hand, in 66% (112/169) of the ARG-containing samples, at least one gene could be linked to a plasmid or iMGE. The ARGs determined to be on a mobile element (whether plasmid or iMGE) are presented according to species in Table 3. For each bacterial species, and for each ARG, the proportion of samples with the mobile ARG in question, among all positive samples for this ARG are shown in Figure 3. 

**Table 3:** ARGs linked to an integrative mobile genetic element or plasmid, according to ARG-positive bacteria species (n = 169 samples)

Species (number of samples with ARG)	ARG (number of samples with ARG on mobile element)
iMGE^a^	
*Bifidobacterium animalis* (n = 65)	*tet(W)* (n = 62)
*Lactiplantibacillus plantarum* (n = 18)	*tet(M)* (n = 1), *tet(S)* (n = 1)^b^
Plasmid^c^	
*Bifidobacterium animalis* (n = 65)	*Bifidobacterium adolescentis* *rpoB* mutants conferring resistance to rifampicin (n = 30), *Limosilactobacillus reuteri cat-TC* (n = 1), *tet(W)* (n = 40)
*Lactiplantibacillus plantarum* (n = 18)	*ANT(3”)-IIa* (n = 2), *ANT(6)-Ia* (n = 1), *catA8* (n = 1), *ErmB* (n = 1), *lnuA* (n = 2), *msrC* (n = 1), *TEM-1* (n = 1), *TEM-181* (n = 1), *tet(C)* (n = 1), *tet(M)* (n = 5), *tet(S)* (n = 1)
*Lactobacillus delbrueckii* (n = 1)	*TEM-116* (n = 1)
*Lactobacillus helveticus* (n = 2)	*lnuA* (n = 2)
*Lactococcus lactis* (n = 74)	*ErmB* (n = 1), *lmrD* (n = 20), *tet(M)* (n = 1), *tet(S)* (n = 4)
*Levilactobacillus brevis* (n = 1)	*lnuA* (n = 1)
*Streptococcus thermophilus* (n = 4)	*ErmB* (n = 2), *tet(S)* (n = 2)

ARG: antimicrobial resistance gene; iMGE: integrated mobile genetic element.

^a^ iMGE-associated ARGs can be on a plasmid or not.

^b^ The *tet(M)* and *tet(S)* ARGs were present in one sample each.

^c^ ARGs borne by a plasmid can be associated to an iMGE or not.

Bado_rpoB_RIF: *Bifidobacterium adolescentis rpoB* mutants conferring resistance to rifampicin gene; Lreu_cat-TC: *Limosilactobacillus reuteri cat-TC* gene*.*

The proportion is the number of samples containing potentially mobile (linked to iMGE or plasmid) ARGs divided by the total number of samples containing ARGs.

## Discussion

Our study confirms that numerous ARGs are present in probiotic bacterial species constituting the bacteriome of edible products and that many of them are mobile. Thus, the application and intake of certain probiotic bacterial strains could have the potential to contribute to the appearance and spread of AMR.

The proportions of samples containing ARGs among those that we examined varied across bacterial species. Samples of *B. animalis* and *L. lactis* appeared to be relatively richer in ARGs. Nevertheless, in the case of *B. animalis* the high proportion of samples with ARG was underlain by the constant detection rate of two genes, namely *tet(W)* and *B. adolescentis rpoB* mutants conferring resistance to rifampicin. Tetracycline-resistance-encoding *tet(W)* is regularly associated with probiotic *B. animalis* strains [35-37]. Moreover, in certain subspecies, this ARG is considered to be innate, phylogenetically distinct from that of other bacterial species and as having a negligible risk of transfer [35]. At the same time, *tet(W)* is commonly flanked by transposase genes [37]. Interestingly, among our 65 ARG-positive *B. animalis* samples, the majority of *tet(W)*-containing samples (n = 62) had this gene associated with iMGEs that were cTns. The interpretation of the mobility potential of the cTn-associated genes is, however, unsure and would require further investigation. Consequently, the public health implications of these findings are not clear. On the other hand, there were 40 samples with *tet(W)* on plasmids in *B. animalis*. For *L. lactis*, lincosamide-resistance encoded *lmrD* showed the highest rate within the examined samples. This gene, in interaction with *lmrC,* is considered to have notable importance in phenotypically multidrug-resistant (MDR) *L. lactis* strains [38]. Just as in most (51/71) of our *lmrD*-positive samples, *lmrD* has been described as chromosomally encoded [39]. Nevertheless, 20 of our samples were associated with plasmid-borne *lmrD*. The plasmidome of *L. lactis* is highly dynamic and of high economic value [40]. Our findings may have public health relevance and may raise awareness of the potential need for more advanced surveillance measures.

In contrast, no *L. casei* or *L. paracasei* strains contained any ARGs, and in *L. delbrueckii*, *L. helveticus* and *L. brevis*, ARGs were relatively less frequent. As the presence of AMR markers is an undesired trait for any microbe introduced in humans as a potential probiotic [41], these species might be favoured in alimentary products. On the other hand, despite the low ARG numbers and diversity, each ARG detected in the above-mentioned species appears to be plasmid-associated, and as such, potentially transferable.

Compared to species where ARGs seemed more prevalent, and species with little occurrence of ARGs in our analyses, the proportions of ARG-positive samples among *L. plantarum*, *L. mesenteroides* and *S. thermophilus* were intermediary in our study, but for these species, the ARGs appeared more diverse. Whereas genomes from *L. mesenteroides* contained no ARGs associated with mobile-genetic-element (plasmid or iMGE), the resistome of *S. thermophilus* was predicted to be highly mobile. The uptake of plasmids carrying ARGs, including *ErmB*, encoding the macrolide-lincosamide-streptogramin B (MLSB) phenotype is not without precedent for streptococci [42]. The samples with *L. plantarum* were more numerous, which may have led to the highest ARG diversity among the species we examined. All but two *L. plantarum* ARGs (*AAC(6’)Ii* and *eatAv*) were located on mobile genetic elements. Among the 18 ARG-positive samples, bacteria in 17 had ARGs on plasmids and in two samples the ARGs were flanked by iMGEs. Consequently, potential implications for public health associated with the *L. plantarum* strains appears non-negligible.

The ARGs identified overall in the current study may affect the activity of several classes of antibiotics, such as aminoglycosides, cephalosporins, lincosamides, macrolides, monobactams, penams, penems, phenicols, pleuromutilins, rifamycins, streptogramins and tetracyclines. They may be involved in resistance mechanisms against many antibiotics used in human and animal medicine. The World Health Organization (WHO) regularly publishes an updated list of human antimicrobials according to their importance, with in 2019, three categories: (i) Critically Important Antimicrobial (CIA) that are the last resort in the treatment of human disease, (ii) Highly Important Antimicrobial (HIA) and (iii) Important Antimicrobial (IA). The CIAs are further subdivided into high priority CIAs and highest priority CIAs. The latter group, abbreviated as HPCIA, includes cephalosporins (3rd, 4th and 5th generation), glycopeptides, ketolides and macrolides, polymyxins and quinolones [43]. Of the five HPCIA drug groups, we found ARGs that potentially could compromise the effectiveness of two (cephalosporins, ketolides and macrolides). We also found ARGs that have the potential to interfere with the activity of five high priority CIAs (aminoglycosides, monobactams, penams, penems, rifamycins), six HIAs (cephalosporins, lincosamides, penams, phenicols, streptogramins, tetracyclines) and one IA (pleuromutilins). The European Medicines Agency (EMA) also produced a list in 2019 aimed at restricting the veterinary use of antimicrobials that are important for human medicine [44]. The antimicrobials are listed under the categories: Avoid, Restrict, Caution and Prudence. We found ARGs that may confer resistance to three drug groups listed as Avoid (cephalosporins, monobactams, streptogramins), one listed as Restrict (cephalosporins), seven as Caution (rifamycins, aminoglycosides, phenicols, lincosamides, macrolides, pleuromutilins, cephalosporins) and two as Prudence (tetracyclines, penams). In addition, the World Organisation for Animal Health (OIE) has a list of critical antimicrobial agents used in veterinary medicine. The OIE uses three categories; Veterinary Critically Important Antimicrobial Agents (VCIA), Veterinary Highly Important Antimicrobial Agents (VHIA) and Veterinary Important Antimicrobial Agents (VIA) [45]. The ARGs we found can have an effect on six VCIAs (tetracyclines, aminogylocosides, phenicols, macrolides, cephalosporins, penams), four VHIAs (rifamycins, lincosamides, pleuromutilins, cephalosporins) and one VIA (streptogramins). Thus, many important human and animal medicine antibiotics could be affected by the ARGs we detected in probiotic bacterial strains from products for human consumption. Nevertheless, it is important to highlight that the presence of ARGs does not necessarily result in the phenotypical appearance of AMR. Further gene expression studies or phenotypical probes (e.g. the assessment of minimal inhibitory concentration values) would be required to evaluate the expressed AMR traits of the examined probiotic bacteria.

Although we consider our results important in the absence of a similar survey study with such a large sample size and a uniform methodology, we must mention their shortcomings and limitations. Our study relies on retrospective data collection. The samples from which data were retrieved had been initially collected over a large period of time and were not obtained from different areas in a systematic way. Therefore, the results are not representative of any particular geographical location at a given time. Moreover, data obtained were based on the NCBI SRA system, which is quite permissive regarding the completeness of the uploaded metadata of samples. Hence, some detailed information can be missing in the metadata, hindering a more thorough presentation and interpretation of the results. We believe that one of the main problems with the extendibility of our results is that the exact types of isolation sources were not available for all samples. Furthermore, it would be very important to know under which conditions (e.g. medium, temperature) each strain was isolated and cultured. It is also unknown whether any antimicrobial agents were used in the cultures to control competing species. If any antimicrobials were used during the culturing process, subpopulations with ARGs could be propagated. The generalisability of our result would also increase if the age of the cultures from which the sequenced strains were isolated was known. Optimally, in a prospective study, these factors could be controlled. Thus, the noise in the variation between species and isolation sources could be reduced. Nonetheless, our work could raise awareness of the need for controlled prospective studies.

### Conclusions

Our results suggest that some probiotic bacterial species may contain a higher proportion of ARGs, while others may represent a lower proportion. We also observe that a considerable proportion of the ARGs that we identified were mobile. In the European Union [46], there are recommendations with methodological suggestions for the whole genome sequencing analysis of microorganisms in the food chain. However, these recommendations do not provide detailed guidelines for the analysis of the mobilome. Since our results suggest that the prevalence of mobile ARGs might not be negligible, it might be worthy to consider the development of guidelines to monitor these mobile ARGs.

## References

[1] European Centre for Disease Prevention and Control (ECDC) European Food Safety Authority (EFSA). European Medicines Agency (EMA). Third joint inter-agency report on integrated analysis of consumption of antimicrobial agents and occurrence of antimicrobial resistance in bacteria from humans and food-producing animals in the EU/EEA: JIACRA III 2016-2018. EFSA J. 2021;19(6):e06712. PMID:34221148 34221148 10.2903/j.efsa.2021.6712 PMC8243991

[2] United Nations, Interagency Coordination Group on Antimicrobial Resistance (IACG). No time to wait: securing the future from drug-resistant infections. Report to the Secretary-General of the United Nations. 2019 [Accessed 12 Jan 2023]. Available from: https://www.who. int/docs/default-source/documents/no-time-to-wait-securing-the-future-from-drug-resistant-infections-en.pdf

[3] Partridge SR Kwong SM Firth N Jensen SO. Mobile Genetic Elements Associated with Antimicrobial Resistance. Clin Microbiol Rev. 2018;31(4):e00088-17.http://dx.doi.org/10.1128/CMR.00088-17 PMID:30068738 30068738 10.1128/CMR.00088-17 PMC6148190

[4] Thomas CM Nielsen KM. Mechanisms of, and barriers to, horizontal gene transfer between bacteria. Nat Rev Microbiol. 2005;3(9):711-21.http://dx.doi.org/10.1038/nrmicro1234 PMID:16138099 16138099 10.1038/nrmicro1234

[5] Todar K. Todars Online Textbook of Bacteriology. Bacterial Resistance to Antibiotics (2020).

[6] Johansson MHK Bortolaia V Tansirichaiya S Aarestrup FM Roberts AP Petersen TN. Detection of mobile genetic elements associated with antibiotic resistance in *Salmonella enterica* using a newly developed web tool: MobileElementFinder. J Antimicrob Chemother. 2021;76(1):101-9.http://dx.doi.org/10.1093/jac/dkaa390 PMID:33009809 33009809 10.1093/jac/dkaa390 PMC7729385

[7] Sharma P Tomar SK Goswami P Sangwan V Singh R. Antibiotic resistance among commercially available probiotics. Food Res Int. 2014;57:176-95.http://dx.doi.org/10.1016/j.foodres.2014.01.025

[8] Zheng M Zhang R Tian X Zhou X Pan X Wong A. Assessing the risk of probiotic dietary supplements in the context of antibiotic resistance. Front Microbiol. 2017;8:908.http://dx.doi.org/10.3389/fmicb.2017.00908 PMID:28579981 28579981 10.3389/fmicb.2017.00908 PMC5437161

[9] Berreta A Baumgardner RM Kopper JJ. Evaluation of commercial veterinary probiotics containing enterococci for transferrable vancomycin resistance genes. BMC Res Notes. 2020;13(1):275.http://dx.doi.org/10.1186/s13104-020-05114-1 PMID:32498700 32498700 10.1186/s13104-020-05114-1 PMC7271421

[10] Rozman V Mohar Lorbeg P Accetto T Bogovič Matijašić B. Characterization of antimicrobial resistance in lactobacilli and bifidobacteria used as probiotics or starter cultures based on integration of phenotypic and *in silico* data. Int J Food Microbiol. 2020;314:108388.http://dx.doi.org/10.1016/j.ijfoodmicro.2019.108388 PMID:31707173 31707173 10.1016/j.ijfoodmicro.2019.108388

[11] Selvin J Maity D Sajayan A Kiran GS. Revealing antibiotic resistance in therapeutic and dietary probiotic supplements. J Glob Antimicrob Resist. 2020;22:202-5.http://dx.doi.org/10.1016/j.jgar.2020.02.007 PMID:32084605 32084605 10.1016/j.jgar.2020.02.007

[12] Tóth AG Csabai I Krikó E Tőzsér D Maróti G Patai ÁV Antimicrobial resistance genes in raw milk for human consumption. Sci Rep. 2020;10(1):7464.http://dx.doi.org/10.1038/s41598-020-63675-4 PMID:32366826 32366826 10.1038/s41598-020-63675-4 PMC7198526

[13] Tóth AG Csabai I Maróti G Jerzsele Á Dubecz A Patai ÁV A glimpse of antimicrobial resistance gene diversity in kefir and yoghurt. Sci Rep. 2020;10(1):22458.http://dx.doi.org/10.1038/s41598-020-80444-5 PMID:33384459 33384459 10.1038/s41598-020-80444-5 PMC7775456

[14] Tóth AG Csabai I Judge MF Maróti G Becsei Á Spisák S Mobile antimicrobial resistance genes in probiotics. Antibiotics (Basel). 2021;10(11):1287.http://dx.doi.org/10.3390/antibiotics10111287 PMID:34827225 34827225 10.3390/antibiotics10111287 PMC8614787

[15] Eloe-Fadrosh EA Brady A Crabtree J Drabek EF Ma B Mahurkar A Functional dynamics of the gut microbiome in elderly people during probiotic consumption. MBio. 2015;6(2):e00231-15.http://dx.doi.org/10.1128/mBio.00231-15 PMID:25873374 25873374 10.1128/mBio.00231-15 PMC4453556

[16] Suez J Zmora N Segal E Elinav E. The pros, cons, and many unknowns of probiotics. Nat Med. 2019;25(5):716-29.http://dx.doi.org/10.1038/s41591-019-0439-x PMID:31061539 31061539 10.1038/s41591-019-0439-x

[17] Montassier E Valdés-Mas R Batard E Zmora N Dori-Bachash M Suez J Probiotics impact the antibiotic resistance gene reservoir along the human GI tract in a person-specific and antibiotic-dependent manner. Nat Microbiol. 2021;6(8):1043-54.http://dx.doi.org/10.1038/s41564-021-00920-0 PMID:34226711 34226711 10.1038/s41564-021-00920-0 PMC8318886

[18] Gueimonde M Delgado S Mayo B Ruas-Madiedo P Margolles A de los Reyes-Gavilán CG. Viability and diversity of probiotic *Lactobacillus* and *Bifidobacterium* populations included in commercial fermented milks. Food Res Int. 2004;37(9):839-50.http://dx.doi.org/10.1016/j.foodres.2004.04.006

[19] Witthuhn R Schoeman T Britz T. Characterisation of the microbial population at different stages of kefir production and kefir grain mass cultivation. Int Dairy J. 2005;15(4):383-9.http://dx.doi.org/10.1016/j.idairyj.2004.07.016

[20] Bourrie BC Willing BP Cotter PD. The microbiota and health promoting characteristics of the fermented beverage kefir. Front Microbiol. 2016;7:647.http://dx.doi.org/10.3389/fmicb.2016.00647 PMID:27199969 27199969 10.3389/fmicb.2016.00647 PMC4854945

[21] Bengoa AA Iraporda C Garrote GL Abraham AG. Kefir micro-organisms: their role in grain assembly and health properties of fermented milk. J Appl Microbiol. 2019;126(3):686-700.http://dx.doi.org/10.1111/jam.14107 PMID:30218595 30218595 10.1111/jam.14107

[22] Van Wyk J. Kefir: The champagne of fermented beverages. In Grumezescu, M. & Holban, A. M. (eds.) *Fermented Beverages*. Duxford: Woodhead Publishing; 2019. 473-527.

[23] Leech J Cabrera-Rubio R Walsh AM Macori G Walsh CJ Barton W Fermented-food metagenomics reveals substrate-associated differences in taxonomy and health-associated and antibiotic resistance determinants. mSystems. 2020;5(6):e00522-20.http://dx.doi.org/10.1128/mSystems.00522-20 PMID:33172966 33172966 10.1128/mSystems.00522-20 PMC7657593

[24] Langmead B Salzberg SL. Fast gapped-read alignment with Bowtie 2. Nat Methods. 2012;9(4):357-9.http://dx.doi.org/10.1038/nmeth.1923 PMID:22388286 22388286 10.1038/nmeth.1923 PMC3322381

[25] Li D Liu C-M Luo R Sadakane K Lam T-W. MEGAHIT: an ultra-fast single-node solution for large and complex metagenomics assembly via succinct de Bruijn graph. Bioinformatics. 2015;31(10):1674-6.http://dx.doi.org/10.1093/bioinformatics/btv033 PMID:25609793 25609793 10.1093/bioinformatics/btv033

[26] Hyatt D Chen GL Locascio PF Land ML Larimer FW Hauser LJ. Prodigal: prokaryotic gene recognition and translation initiation site identification. BMC Bioinformatics. 2010;11(1):119.http://dx.doi.org/10.1186/1471-2105-11-119 PMID:20211023 20211023 10.1186/1471-2105-11-119 PMC2848648

[27] McArthur AG Waglechner N Nizam F Yan A Azad MA Baylay AJ The comprehensive antibiotic resistance database. Antimicrob Agents Chemother. 2013;57(7):3348-57.http://dx.doi.org/10.1128/AAC.00419-13 PMID:23650175 23650175 10.1128/AAC.00419-13 PMC3697360

[28] Jia B Raphenya AR Alcock B Waglechner N Guo P Tsang KK CARD 2017: expansion and model-centric curation of the comprehensive antibiotic resistance database. Nucleic Acids Res. 2017;45(D1):D566-73.http://dx.doi.org/10.1093/nar/gkw1004 PMID:27789705 27789705 10.1093/nar/gkw1004 PMC5210516

[29] Buchfink B Xie C Huson DH. Fast and sensitive protein alignment using DIAMOND. Nat Methods. 2015;12(1):59-60.http://dx.doi.org/10.1038/nmeth.3176 PMID:25402007 25402007 10.1038/nmeth.3176

[30] Krawczyk PS Lipinski L Dziembowski A. PlasFlow: predicting plasmid sequences in metagenomic data using genome signatures. Nucleic Acids Res. 2018;46(6):e35-35.http://dx.doi.org/10.1093/nar/gkx1321 PMID:29346586 29346586 10.1093/nar/gkx1321 PMC5887522

[31] Guo J Bolduc B Zayed AA Varsani A Dominguez-Huerta G Delmont TO VirSorter2: a multi-classifier, expert-guided approach to detect diverse DNA and RNA viruses. Microbiome. 2021;9(1):37.http://dx.doi.org/10.1186/s40168-020-00990-y PMID:33522966 33522966 10.1186/s40168-020-00990-y PMC7852108

[32] Stevenson, M. *et al.* *epiR: Tools for the Analysis of Epidemiological Data* (2022). R package version 2.0.54.

[33] R Core Team. *R: A Language and Environment for Statistical Computing*. R Foundation for Statistical Computing, Vienna, Austria (2021).

[34] Lokesh D Parkesh R Kammara R. *Bifidobacterium adolescentis* is intrinsically resistant to antitubercular drugs. Sci Rep. 2018;8(1):11897.http://dx.doi.org/10.1038/s41598-018-30429-2 PMID:30093677 30093677 10.1038/s41598-018-30429-2 PMC6085307

[35] Nøhr-Meldgaard K Struve C Ingmer H Agersø Y. The tetracycline resistance gene, *tet(W)* in *Bifidobacterium animalis* subsp. *lactis* follows phylogeny and differs from *tet(W)* in other species. Front Microbiol. 2021;12:658943.http://dx.doi.org/10.3389/fmicb.2021.658943 PMID:34335493 34335493 10.3389/fmicb.2021.658943 PMC8319848

[36] Ammor MS Flórez AB Alvarez-Martín P Margolles A Mayo B. Analysis of tetracycline resistance *tet(W)* genes and their flanking sequences in intestinal *Bifidobacterium* species. J Antimicrob Chemother. 2008;62(4):688-93.http://dx.doi.org/10.1093/jac/dkn280 PMID:18614524 18614524 10.1093/jac/dkn280

[37] Gueimonde M Flórez AB van Hoek AH Stuer-Lauridsen B Strøman P de los Reyes-Gavilán CG Genetic basis of tetracycline resistance in *Bifidobacterium animalis* subsp. *lactis.* Appl Environ Microbiol. 2010;76(10):3364-9.http://dx.doi.org/10.1128/AEM.03096-09 PMID:20348299 20348299 10.1128/AEM.03096-09 PMC2869156

[38] Lubelski J de Jong A van Merkerk R Agustiandari H Kuipers OP Kok J *LmrCD* is a major multidrug resistance transporter in *Lactococcus lactis.* Mol Microbiol. 2006;61(3):771-81.http://dx.doi.org/10.1111/j.1365-2958.2006.05267.x PMID:16879641 16879641 10.1111/j.1365-2958.2006.05267.x

[39] Flórez AB de Los Reyes-Gavilán CG Wind A Mayo B Margolles A. Ubiquity and diversity of multidrug resistance genes in *Lactococcus lactis* strains isolated between 1936 and 1995. FEMS Microbiol Lett. 2006;263(1):21-5.http://dx.doi.org/10.1111/j.1574-6968.2006.00371.x PMID:16958846 16958846 10.1111/j.1574-6968.2006.00371.x

[40] Ainsworth S Stockdale S Bottacini F Mahony J van Sinderen D. The *Lactococcus lactis* plasmidome: much learnt, yet still lots to discover. FEMS Microbiol Rev. 2014;38(5):1066-88.http://dx.doi.org/10.1111/1574-6976.12074 PMID:24861818 24861818 10.1111/1574-6976.12074

[41] EFSA Panel on Additives and Products or Substances used in Animal Feed (FEEDAP). Guidance on the assessment of bacterial susceptibility to antimicrobials of human and veterinary importance. EFSA J. 2012;10:2740.

[42] Li L Olsen RH Shi L Ye L He J Meng H. Characterization of a plasmid carrying cat, *ermB* and *tetS* genes in a foodborne *Listeria monocytogenes* strain and uptake of the plasmid by cariogenic *Streptococcus mutans.* Int J Food Microbiol. 2016;238:68-71.http://dx.doi.org/10.1016/j.ijfoodmicro.2016.08.038 PMID:27592072 27592072 10.1016/j.ijfoodmicro.2016.08.038

[43] World Health Organization (WHO) Advisory Group on Integrated Surveillance of Antimicrobial Resistance (AGISAR). WHO list of critically important antimicrobials for human medicine (WHO CIA list). Tech. Rep. Geneva: WHO; 2019. [Accessed 12 Jan 2023]. Available from: https://www.who.int/publications/i/item/9789241515528

[44] European Medicines Agency (EMA). Categorisation of antibiotics for use in animals for prudent and responsible use (2019). [Accessed 12 Jan 2023]. Amsterdam: EMA. Available from: https://www.ema.europa.eu/en/news/categorisation-antibiotics-used-animals-promotes-responsible-use-protect-public-animal-health

[45] World Organisation for Animal Health. (OIE, WOFAH). OIE list of antimicrobial agents of veterinary importance. J. OIE Int. Commit. 2015;33:1-9.

[46] European Food Safety Authority (EFSA). EFSA statement on the requirements for whole genome sequence analysis of microorganisms intentionally used in the food chain. EFSA J. 2021;19(7):e06506. PMID:34335919 34335919 10.2903/j.efsa.2021.6506 PMC8317053

